# Inhaled ciclesonide versus inhaled budesonide or inhaled beclomethasone or inhaled fluticasone for chronic asthma in adults: a systematic review

**DOI:** 10.1186/1471-2296-7-34

**Published:** 2006-06-05

**Authors:** Matthew J Dyer, David MG Halpin, Ken Stein

**Affiliations:** 1 Clinical Research Assistant, Peninsula Medical School, University of Exeter, UK; 2 Consultant Physician & Senior Lecturer in Respiratory Medicine, Royal Devon and Exeter Hospital, Exeter, UK; 3 Senior Clinical Lecturer in Public Health, Peninsula Medical School, University of Exeter, UK

## Abstract

**Background:**

Ciclesonide is a new inhaled corticosteroids licensed for the prophylactic treatment of persistent asthma in adults. Currently beclomethasone dipropionate, budesonide and fluticasone propionate are the most commonly prescribed inhaled corticosteroids for the treatment of asthma but there has been no systematic review comparing the effectiveness and safety ciclesonide to these agents. We therefore aimed to systematically review published randomised controlled trials of the effectiveness and safety of ciclesonide compared to alternative inhaled corticosteroids in people with asthma.

**Methods:**

We performed literature searches on MEDLINE, EMBASE, PUBMED, the COCHRANE LIBRARY and various Internet evidence sources for randomised controlled trials or systematic reviews comparing ciclesonide to beclomethasone or budesonide or fluticasone in adult humans with persistent asthma. Data was extracted by one reviewer.

**Results:**

Five studies met the inclusion criteria. Methodological quality was variable. There were no trials comparing ciclesonide to beclomethasone. There was no significant difference between ciclesonide and budesonide or fluticasone on the following outcomes: lung function, symptoms, quality of life, airway responsiveness to a provoking agent or inflammatory markers. However, the trials were very small in size, increasing the possibility of a type II error. One trial demonstrated that the combined deposition of ciclesonide (and its active metabolite) in the oropharynx was 47% of that of budesonide while another trial demonstrated that the combined deposition of ciclesonide (and its active metabolite) in the oropharynx was 53% of that of fluticasone. One trial demonstrated less suppression of cortisol in overnight urine collection after ciclesonide compared to fluticasone (geometric mean fold difference = 1.5, P < 0.05) but no significant difference in plasma cortisol response.

**Conclusion:**

There is very little evidence comparing CIC to other ICS, restricted to very small, phase II studies of low power. These demonstrate CIC has similar effectiveness and efficacy to FP and BUD (though equivalence is not certain) and findings regarding oral deposition and HPA suppression are inconclusive. There is no direct comparative evidence that CIC causes fewer side effects since none of the studies reported patient-based outcomes.

## Background

Inhaled corticosteroids (ICS) have a central role in the treatment of asthma. They are the most effective prophylactic agents available, particularly in patients with mild to moderate asthma and persistent symptoms[1] and are recommended for most adult patients with chronic asthma whose symptoms are not controlled by inhaled short acting β 2 agonists [2-4]. Regular treatment with corticosteroids reduces exacerbations, improves control of symptoms and lung function, while reducing hospital admissions and deaths from asthma[1,5]. However, prolonged use in persistent asthma and increased doses in severe cases may result in suppression of the hypothalamic pituitary adrenal (HPA) system with concern that this might cause growth impairment in children (including premature closure of the epiphyses of long bones), disturbed glucose tolerance, decreased mineralisation of bone (increasing the risk of fractures), ocular problems such as glaucoma and cataracts as well as thinning of the skin [6-8]. Local adverse affects, even at low doses may include dysphonia, pharyngitis and oral candidiasis[2,9].

The mainstay of ICS treatment has been with three agents: beclomethasone dipropionate (BDP), budesonide (BUD) and, more recently, fluticasone propionate (FP). All these agents are similar chemically and structurally but have different pharmacodynamic properties resulting in different clinical effects[10]. A Cochrane review of 48 studies comparing the three agents concluded that FP given at half the daily dose of BDP or BUD leads to small improvement in measures of airway calibre (peak expiratory flow (PEF) and forced expiratory volume in one second (FEV1) while at the same daily dose FP appears to have a higher risk of side effects than BDP or BUD[10].

Ciclesonide (CIC) is a new ICS, manufactured by Altana Pharma Ltd. CIC is licensed only for the treatment of persistent asthma in adults (18 years older) and is delivered via a hydrofluoroalkane metered-dose inhaler (HFA MDI) in 40, 80 and 160 mcg formulations. The recommended starting dose is 160 mcg given in the evening, with reduction to 80 mcg for maintenance[11]. These doses are "ex-actuator", i.e. the dose expelled, as opposed to "ex-valve" i.e. the actual dose contained in the inhaler (for consistency, throughout this review ex-valve doses are used for all inhalers).

Ciclesonide has little anti-inflammatory activity itself and requires cleavage by endogenous carboxyl esterases in the lung, which creates the active metabolite desisobutryl-ciclesonide (des-CIC)[12]. This targets activity at the desired location. Des-Ciclesonide undergoes rapid hepatic metabolism into inactive metabolites on leaving the lung[13]. These factors, together with the fact that ciclesonide has very low oral bioavailability due to almost complete first pass metabolism[14] would seem to create conditions favouring the maximisation of therapeutic effect in the lung and minimisation of the risk of systemic adverse effects.

Given that ciclesonide is being actively marketed as an alternative to alternative to other inhaled corticosteroids, our objective in this study was to systematically review published randomised controlled trials of the effectiveness and safety of ciclesonide compared to alternative inhaled corticosteroids in people with asthma.

## Methods

### Search strategy

We performed literature searches on MEDLINE (from 1951), EMBASE, PUBMED and the COCHRANE LIBRARY (The Cochrane Central Register of Controlled Trials) using the following terms:

Ciclesonide, Alvesco, Budesonide, Pulmicort, Beclomethasone, Becotide, Becloforte, Fluticasone, Flixotide

We imported abstracts of citations from this search into an electronic database. We also searched for "ciclesonide" and "alvesco" on the web sites of the following internet evidence sources:

• Drug and Therapeutics Bulletin 

• Succinct and Timely Evaluated Evidence Reviews (STEER)

• Aggressive Research Intelligence Facility 

• West Midlands Health Technology Assessment Collaboration 

• International Network of Agencies for Health Technology Assessment 

• Canadian Coordinating Office for Health Technology Assessment 

Only the CCOHTA site yielded a result: a non-systematic review. This was subsequently excluded.

Reference lists of retrieved articles were scrutinized for further studies but yielded no additional papers.

Titles and abstracts were sifted by two reviewers and prior to retrieval of full articles, the two reviewers independently assessed papers for inclusion. Cases of disagreement were resolved by discussion (or, where necessary, delayed until retrieval of the full text). All full text papers retrieved were again assessed by the two independent reviewers for inclusion and methodological quality (see Appendix 1). There was no blinding to authors' names or institutions and no scoring system (such as the Jadad score) was used. Data were extracted by one reviewer.

### Inclusion criteria for considering studies

#### Participants

Studies in human adults (i.e. people aged 18 and over) with a diagnosis of chronic asthma were included. We did not consider studies concerning acute asthma, chronic obstructive pulmonary disease or allergic rhinitis.

#### Intervention

Studies concerning only inhalation of ciclesonide and excluded those involving oral, nasal or intravenous routes.

#### Comparators

Studies that compared ciclesonide to either budesonide or beclomethasone or fluticasone and excluded studies comparing to placebo only or to other asthma treatments.

#### Outcome measures

we considered all reported outcomes although prominence was given to patient based outcomes.

#### Study design

Prospective, randomized, controlled trials and or reviews that were clearly systematic and carried out since the beginning of 2004. Studies published only as abstracts were included only if they contained sufficient methodological detail to enable critical appraisal. We considered studies in all languages.

## Results

### Search results

Table 1 shows the results of the search. A total of five RCTs were included (details in Table 2) [15-19]. No reviews or abstracts qualified for inclusion. Two RCTs compared CIC to BUD[15,19] and three RCTs compared CIC to FP [16-18]. The Kanniess *et al *study[19] was the only RCT not apparently sponsored by a pharmaceutical company manufacturing CIC.

**Table 1:** Search Results Summary

Search	Medline	Embase	PubMed	Cochrane	Internet	Total no. of papers without duplicates	Reviews	Abstracts	Papers after exclusion
(Ciclesonide or Alvesco) and (Budesonide or Pulmicort)	6	58	7	7	1	66	4 (2 are duplicates with the FP search)	5	2
(Ciclesonide or Alvesco) and (Beclomethasone or Becotide or Becloforte)	1	1	1	0	0	2	0	0	0
(Ciclesonide or Alvesco) and (Fluticasone or Flixotide)	4	56	4	4	0	62	2 (both are duplicates with the FP search)	3	3

**Table 2:** RCTs included in the review

Author	Publisher	Sponsor	Study size	Duration	Comparator
Nave *et al*, 2005	European Journal of Clinical Pharmacology	ALTANA	18	Measurements at 0, 15, 30, 45 and 60 mins	CIC 800 mcg (HFA MDI) od am Vs. BUD 800 mcg (turbohaler) od am
Richter *et al*, 2005	Journal of Clinical Pharmacology	ALTANA	18	Measurements at 0, 15, 30, 45 and 60 mins	CIC 800 mcg (HFA MDI) od am Vs. FP 1000 mcg (HFA MDI) od am
Lee, Fardon *et al*, 2005	Chest	AVENTIS	14	4 weeks Crossover with 2 week washout period	CIC 800 mcg (HFA MDI) bd Vs. FP 1000 mcg (HFA MDI) bd
Lee, Haggart *et al*, 2004	British Journal of Clinical Pharmacology	AVENTIS	19	4 weeks Crossover with 2 week washout period	CIC 400 mcg (HFA MDI) od am Vs. FP 250 mcg (HFA MDI) bd
Kanniess *et al*, 2001	Pulmonary Pharmacology and Therapeutics	None declared	15	2 weeks (Cross over study with at least 3 week washout period)	CIC 400 mcg (HFA MDI) od am Vs. BUD 400 mcg (turbohaler) od am

### Quality of the evidence

All papers were critically appraised for methodological quality based on the criteria shown in Table 3. All trials involved small numbers (the largest number of participants completing the study being 19[18]) and were of very short duration (maximum four weeks). Three of the studies had drop-outs after randomization with attrition rates varying from 5.25% – 30% [17-19]. In general, populations were similar, although two studies did not exclude smokers. Participants had mild asthma with mean FEV1 greater than 90% of the predicted value in three of the trials.

**Table 3:** RCT Methodological Characteristics

**Author**	**Year**	**Population**		**Comp.**	**Duration**	**Conceal-ment**	**Blinding**		**Random-isation**	**Attrition**	**Balance at baseline**	**Equal handling**
Kanniess *et al*	2001	No. completing	15	BUD	2 weeks crossover 3–8 weeks washout	No info about allocation	Participant	Yes	Yes but no evidence of method	1 (5.25%)	no	yes
		Mean age	33				Admin.	no				
		Stable	yes				Observer	yes				
		Non smokers only	yes				Analyst	?				
		Mean FEV1 % pred:	94									

Nave *et al*	2005	No. completing	18	BUD	Combined treatment Oropharyngeal washings taken at 0 mins on day 1, 15 mins on day2, 30 mins on day 3, 45 mins on day 4 and 60 mins on day 5.	No info about allocation	Participant	no	Yes but no evidence of method	0	yes	yes
		Mean age	33				Admin.	no				
		Stable	yes				Observer	no				
		Non smokers only	no				Analyst	?				
		Mean FEV1 % pred:	?									

Lee, Fardon *et al*	2005	No. completing	14	FP	4 weeks crossover 2 weeks washout	No info about allocation	Participant	yes	Yes but no evidence of method	6 (30%)	yes	yes
		Mean age	47				Admin.	?				
		Stable	yes				Observer	?				
		Non smokers only	yes				Analyst	?				
		Mean FEV1 % pred:	77									

Lee, Haggart *et al*	2004	No. completing	19	FP	4 weeks crossover 2 weeks washout	No info about allocation	Participant	yes	Yes but no evidence of method	4 (17.5%)	yes	yes
		Mean age	45				Admin.	?				
		Stable	yes				Observer	?				
		Non smokers only	yes				Analyst	?				
		Mean FEV1 % pred:	90									

Richter *et al*	2005	No. completing	18	FP	Combined treatment Treatement 5–14 days (5 treatments in total at 0, 15, 30, 45 and 60 minutes). Minimum 1 day washout	No info about allocation	Participant	no	Yes but no evidence of method	0	yes	yes
		Mean age	37				Admin.	no				
		Stable	yes				Observer	no				
		Non smokers only	no				Analyst	?				
		Mean FEV1 % pred:	91									

Three of the studies were cross-over trials and washout periods were all of appropriate length [17-19]. Nave *et al *and Richter *et al *were" within patient" trials, and therefore similar to a crossover design, but both treatments were given concurrently, presumably on the assumption that oropharyngeal deposition was independent of treatment but potentially confounded by short term changes in oropharyngeal conditions. There were no parallel studies. There was insufficient reporting to verify whether there was good balance at baseline in the crossover trials. In the Kanniess *et al *study there was also imbalance between the population as a whole before receiving CIC and the same population before receiving BUD.

Only one trial (Kanniess *et al*) was clearly double (or more) blinded. Lee, Fardon *et al *and Lee, Haggart *et al *it state that the inhalers were "masked" but it is not clear whether their identity was withheld from the treatment administrator or observer as well as the patient. None of the trials reported whether, or how, they concealed allocation of treatment within participants or methods for randomization. Overall there was no evidence of performance bias.

Only two trials (Lee, Fardon *et al *and Lee, Haggert *et al*) measured patient based outcomes as end points i.e. symptoms and QoL. All the others measured intermediate outcomes and any interpretation of these results will require an assessment of the degree to which these outcomes are clinically significant.

Some studies (e.g. Kanniess *et al *[19]and to some extent Lee, Fardon *et al*[17]) only reported pre and post treatment results within the same treatment (i.e. CIC or the comparator). Although the authors stated there was no significant difference between treatments, they showed no data or calculations.

### Outcomes measured in the trials

Lung function tests

• FEV1, FVC, PEF etc.

Symptoms

• Symptom diary

• Use of rescue medication

Quality of Life

• Mini Asthma quality of life (QoL) questionnaire[20]

Airway responsiveness to provoking agent

• This is measured in terms of the concentration of inhaled provoking agent (adenosine monophosphate (AMP) or metacholine) required to cause a 20% fall in FEV1 (PC20). The initial dose is inhaled and the FEV1 measured subsequently. The dose is then doubled progressively until a 20% fall is recorded

Inflammatory markers

• Nitric oxide (NO) exhaled

• Inflammatory markers in the sputum

Hypothalamic-Pituitary-Adrenal (HPA) suppression (systemic toxicity)

• Plasma cortisol response to human corticotrophin-releasing factor (hCRF). This test has been shown to detect impaired adrenal reserves in corticosteroid-treated patients[21].

• Urine cortisol

Oropharyngeal deposition

• This is the amount of inhaled steroid that does not enter the lung and is deposited in the oropharynx.

Table 4 shows the comparative results from the trials.

**Table 4:** Summary of Results from RCTs Comparing Ciclesonide to Budesonide or Fluticasone

**Trial**	**Results CIC vs. comparator**				
**Lung Function**					

Lee, Fardon *et al*	Authors report no significant difference (CIC vs. FP)				
Lee, Haggart *et al*		95% CI for CIC vs. FP			
	FEV1 (l)	-0.15, 0.06			
Kanniess *et al*	Authors report no significant difference (CIC vs. BUD)				

**Patient Symptoms**					

Lee, Fardon *et al*	Authors report no significant difference (CIC vs. FP)				
Lee, Haggart *et al*					
		95% CI for CIC vs. FP			
	PEF (am) (l/min)	-12, 14			
	PEF (pm) (l/min)	-11. 17			
	Asthma symptom score (am)	-0.3, 0.1			
	Asthma symptom score (pm)	-0.3, 0.1			
	Rescue (am) (puffs/day)	-0.4, 0.2			
	Rescue (am) (puffs/day)	-0.3, 0.1			

**Quality of Life**					

Lee, Fardon *et al*	Authors report no significant difference (CIC vs. FP)				
Lee, Haggart *et al*					
		95% CI for CIC vs. FP			
	Activities	-0.26, 0.92			
	Symptoms	-0.45, 0.56			
	Emotions	-0.76, 0.37			
	Environment	-1.08, 0.02			
	Overall	-0.43, 0.37			

**Airway Responsiveness to Provoking Agent**					

Lee, Fardon *et al*					
		CIC vs. FP			
		GMFD	95% CI	P	
	PC20 FEV1 (metacholine)	0.1	-0.7-0.5	>0.05	
Lee, Haggart *et al*					
		95% CI for CIC vs. FP			
	PC20 FEV1 (metacholine)	-1.2, 0.4			
Kanniess *et al*	Authors report no significant difference (CIC vs. BUD)				

**Inflammatory Markers**					

Lee, Fardon *et al*					
		CIC vs. FP			
		GMFD	95% CI	P	
	Exhaled Nitric oxide	1.4	0.8–2.5	>0.05	
Lee, Haggart *et al*					
		95% CI for CIC vs. FP			
	Exhaled Nitric oxide	-2.1, 7.3			
Kanniess *et al*	Authors report no significant difference (CIC vs. BUD)				

**HPA Suppression**					

Lee, Fardon *et al*					
		CIC vs. FP			
		GMFD	95% CI	P	
	Cortisol pre-hCRF	1.1	0.9–1.2	>0.05	
	Cortisol 30 mins post-hCRF	1.0	0.9–1.2	>0.05	
	OUC	1.5	1.1–2.0	<0.05	

**Oropharyngeal Deposition**					

Nave *et al*	Test	Reference	Point Estimates of Molar Adjusted AUC0–60 min Ratios	95% CI	P value
	des-CIC	BUD	0.04	0.02 – 0.05	< 0.0001
	des-CIC	CI C	0.08	0.06 – 0.11	< 0.0001
	des-CIC + CIC	BUD	0.47	0.38 – 0.59	< 0.0001
Richter *et al*					
	Test	Reference	Point Estimates of Molar Adjusted AUC0–60 min Ratios	95% CI	P value
	des-CIC	FP	0.08	0.05 – 0.11	<0.00001
	des-CIC	CIC	0.17	0.13 – 0.22	<0.00001
	des-CIC + CIC	FP	0.53	0.40 – 0.69	<0.001

### Results from the trials

None of the trials showed CIC to have any benefit over either FP or BUD for the outcomes of lung function, symptoms, quality of life, airway responsiveness to a provoking agent or inflammatory markers.

Lee, Fardon *et al *studied HPA suppression. At the end of each four week treatment period, of either CIC 800 mcg bd or FP 1000 mcg bd, a 10 hour overnight urine collection (OUC) was taken and plasma cortisol response to a 100 mcg bolus of hCRF was assessed at 30 and 60 minutes. The authors state that data were logarithmically transformed to normalize the distribution but give no comment on how the data were skewed. Results comparing CIC and FP are reported as the geometric mean fold difference (GMFD) but there is no explanation as to how these values were calculated. By definition a GMFD of 1.0 means no difference.

When comparing the two treatments there was no significant difference in outcome with respect to plasma cortisol response to hCRF. However, there was significantly more suppression of urinary cortisol after FP than CIC (but with 95% CI of 1.1–2.0 this was only barely so). The results for plasma cortisol levels 60 minutes after hCRF are not reported in the table but the authors state that there was no significant difference between pretreatment and post treatment FP levels.

Two trials studied orpharyngeal deposition and were very similar in nature[15,16]. Nave *et al *compared CIC 800 mcg via a hydrofluoroalkane-pressurised metered-dose inhaler (HFA MDI) to BUD 800 mcg via a chlorofluorocarbon-pressurised metered dose inhaler (CFC MDI). Richter *et al *compared CIC 800 mcg to FP 1000 mcg each via HFA MDI. Curves were plotted for recovery of each drug in rinsing solution against time after administration and then the molar area under the curve for 0 – 60 minutes (AUC_0–60 min_) was calculated for CIC, des-CIC and BUD (or FP) to allow direct comparisons.

The Nave *et al *study shows that the combined deposition of CIC and des-CIC in the oropharynx was less than half (47%) of that of BUD. Only 8% of the CIC deposited was converted into the active metabolite des-CIC (suggesting a lack of converting esterases in the oropharynx). Overall the concentration of des-CIC in the oropharynx 60 minutes after inhalation was only 4% of the BUD concentration (i.e. 25 times more BUD than des-CIC).

The Richter *et al *study shows that the combined deposition of CIC and des-CIC in the oropharynx was only 53% of that of FP. Furthermore only 17% of the CIC deposited was converted into the active metabolite des-CIC. The concentration of des-CIC in the oropharynx 60 minutes after inhalation was only 8% of the FP concentration (i.e. 12.5 times more FP than des-CIC).

## Discussion

There are few data directly comparing CIC to other ICS and no published evidence directly comparing CIC to BDP specifically. None of the RCTs showed CIC to offer any benefit over BUD or FP for effectiveness i.e. none of the RCTs showed CIC to offer any benefit over BUD or FP for any patient based outcomes (asthma symptoms or QoL in these trials). Furthermore none of the trials demonstrated any benefit from CIC over BUD or FP for indirect outcomes of efficacy i.e. lung function, improving response to AMP or metacholine as provoking agents or for decreasing markers of inflammation.

All but one of the trials were sponsored by drug companies manufacturing CIC and seem to endeavour to demonstrate CIC to have equivalent efficacy to other ICS but with an improved safety profile. However, none of the studies report analyses which exclude superiority of one treatment over another (hence it is not possible to conclude that CIC was equivalent to FP or BUD for any efficacy outcomes) and the evidence regarding safety is not conclusive.

The conflicting evidence from the Lee, Fardon *et al *trial might indicate that CIC has less systemic adverse effects than FP. Challenges to this conclusion, however, are twofold. The first comes from the trial itself. This is the only published trial comparing HPA suppression between CIC and other ICS and the results were not unequivocal. There were also some methodological weaknesses in the trial. There was no evidence of concealment of allocation, an attrition rate of 30%, no evidence of blinding other than the participants and the choice of a comparator (i.e. FP) that is reported to have the highest risk of side effects[10] (there is no published evidence directly comparing HPA suppression after treatment with CIC to either BUD or BDP).

The second challenge relates to the correlation between the intermediate outcome of HPA suppression measured at four weeks after the start of treatment with ICS correlates and clinically adverse effects for patients. HPA suppression is a well reported outcome of both short and long term ICS use [22-25]. However the clinical significance of such suppression is uncertain[6,22,25]. Current evidence suggests that ICS do not cause important systemic side effects in doses of up to 400 mcg/day in children and 800 mcg/day in adults,[26] and even in doses of more than 1 mg/day there is no conclusive evidence that patients are at any increased risk from side-effects[25,23]. Hanania *et al *report HPA suppression and decreased bone density after regular use of conventional doses of ICS for asthma[24] but there is no conclusive evidence of a clinically adverse effect e.g. bone fractures.

Further long term studies are required to determine the long term risk of clinically significant adverse effects as a result of HPA suppression associated with ICS use in general and specifically with CIC. In the meantime it is not possible to conclude that CIC offers any benefit over other ICS in terms of systemic adverse effects.

With respect to local adverse effects there are similar challenges. Although, CIC might be expected to have fewer local adverse effects (due to the inhaled agent being its inactive metabolite des-CIC) there is no logical explanation why CIC should be deposited in the oropharynx in such smaller amounts than FP or BUD. In the Nave *et al *study the authors point out that the difference in deposition could be due to the different inhaler devices used. HFA MDIs have been shown to produce ICS with a smaller particle size than CFC MDIs[27] resulting in 17% of a 200 mcg dose of BUD being respirable[15] compared to 48% of a 200 mcg dose of CIC[8,13]. However, in the Richer *et al *study both inhalers were HFA MDI and the authors make no mention of why deposition might be less given that both treatments are inhaled via the same device. Neither trial was blinded in any way, which could have been a source of bias, and the lack of a logical explanation for such vastly different deposition rates makes it difficult to draw any definite conclusion. Further studies (preferably parallel) with larger populations are required before concluding whether CIC offers any benefit in terms of local adverse effects

In addition to the RCTs outlined above there are number of abstracts that have not yet been published as full papers. These trials involved substantially larger numbers of participants and ran for longer duration but have not been included in the analysis since they contained insufficient detail for critical appraisal of methodological quality. The results reported in these abstracts do not alter the conclusions drawn from the full papers but are reported for interest in Appendix 1.

Irrespective of any clinical benefit or not CIC is more expensive compared to BEC, BUD and FP as shown in Table 5. Treatment with CIC would come at substantial financial cost since at high dose (1000 mcg daily) CIC is 5.13 as expensive as BDP, 2.27 times as expensive as BUD (800 mcg daily) and 1.39 times as expensive as FP.

**Table 5:** Cost of Inhaled Corticosteroids at various doses

**ICS**	**Inhaler**	**Ex-valve Daily dose (mcg)**	**Cost for 28 days treatment (€)**
Beclomethasone	MDI	100	1.29
Beclomethasone	MDI	400	2.28
Beclomethasone	MDI	1000	9.16
Budesonide	Turbohaler	100	2.05
Budesonide	Turbohaler	400	10.36
Budesonide	Turbohaler	800	20.72
Fluticasone	HFA MDI	100	2.53
Fluticasone	HFA MDI	500	19.84
Fluticasone	HFA MDI	1000	33.73
Ciclesonide	HFA MDI	100	6.66
Ciclesonide	HFA MDI	400	15.68
Ciclesonide	HFA MDI	1000	47.04

Source: Department of Health Drug Tariff May 2005

Any advantage that CIC might have over existing, cheaper, ICS is predicated on assertions regarding the long term dangers of ICS use. "Steroid phobia" is recognised in other fields[28] and is likely to form the basis for effective direct to patient marketing of CIC, where such advertising is permitted. However, the evidence base on long term inhaled steroid use is far from certain and it is not clear whether the dangers are such that the precautionary principle is justified.

Although it is clear that the evidence base for ciclesonide will expand considerably with the publication of the larger studies excluded from this review, we believe it is important to highlight the limited nature of the evidence base that is currently available for scrutiny by clinicians and policy makers seeking to practice and support evidence based medicine.

## Conclusion

There is very little evidence that has been published in full comparing CIC to other ICS. Current evidence is restricted to very small, phase II studies of low power. These demonstrate CIC has similar effectiveness and efficacy to FP and BUD (though equivalence is not certain) and findings regarding oral deposition and HPA suppression are inconclusive. There is no direct comparative evidence that CIC causes fewer side effects since none of the studies reported patient-based outcomes. Treatment with CIC would also come at substantial financial cost compared to other ICS.

## Competing interests

MD – None

KS – None

DH has received sponsorship to attend international meetings and honoraria for lecturing, attending advisory boards and preparing educational materials from Altana, AstraZeneca, Boehringer Ingelheim, GlaxoSmithKline and Pfizer

## Authors' contributions

KS and MD designed the study. MD performed the searches, retrieved papers, extracted data. MD and KS applied inclusion criteria, carried out the narrative synthesis and drafted the manuscript. DH participated in the design and coordination and helped to draft the manuscript. All authors read and approved the final manuscript. KS is guarantor.

## Funding

No specific funding was received for the study.

MD's post in PenTAG was funded by the NHS Public Health Training Scheme (South West Region).

## Appendix 1

The search resulted in the retrieval of eight abstracts recorded in Table 6. The Hansel *et al *and Engelstatter *et al *abstracts had the same author group, trial characterists and results and were assumed to be from the same trial. Hence only one (Hansel *et al*) was included. The Fardon *et al *abstract appeared to be an abstract form of the Lee, Haggart *et al *full paper and hence the abstract was excluded. The Derom *et al *and Pauwels *et al *abstracts were identical in all ways other than that the former had 25 participants and the latter 26. This could have been a typographical error and they were assumed to be abstracts of the same trial and only the Derom *et al *abstract included. The Biberger *et al *and Ukena *et al *abstracts had exactly the same author group, the same number of trial participants, the same trial and comparator doses but a slight difference in the results i.e. FEV1 increase after CIC and FP was 411 ml and 319 ml respectively in Biberger *et al *and 416 ml and 321 ml respectively in Ukena *et al *with all other results the same. It was assumed that the data had been analysed differently in each case but that these results represented the same trial and only one (Ukena *et al*) was included. Table 7 shows details of the abstracts.

**Table 6:** Initial Retrieval of Trials Published as Abstracts

Author	Date	Publisher	Sponsor	Comparator	Comments
Boulet *et al *[29]	2003	American.Journal of Respiratory.and Critical.Care Medicine	ALTANA	BUD	
Ukena *et al *[30]	2003	European Respiratory Journal	None declared	BUD	Same as Biberger abstract
Engelstatter *et al *[31]	2003	American.Journal of Respiratory.and Critical.Care Medicine	ALTANA	BUD	Same as Hansel abstract
Biberger *et al *[32]	2003	American.Journal of Respiratory.and Critical.Care Medicine	ALTANA	BUD	Same as Ukena abstract
Hansel *et al *[33]	2003	European Respiratory Journal	ALTANA	BUD	Same as Engelstatter abstract
Fardon *et al *[34]	2004	Journal of Allergy and Clinical.Immunology	None declared	FP	Same as Lee, Haggert full paper
Pauwels *et al *[35]	2002	American.Journal of Respiratory.and Critical.Care Medicine	ALTANA	FP	Same as Derom abstract
Derom *et al*[36]	2001	Oral presentation at European Respiratory Society Annual Congress, September 22–26, Berlin, Germany 2001	ALTANA	FP	Same as Pauwels abstract

**Table 7:** Details of Abstracts (duplicates removed)

**Author**	**Date**	**Comparator**	**Study size**	**Reported characterists**	**Duration**	**Outcome Measures**
Boulet *et al*	2003	CIC 400 mcg (mdi) od am Vs. BUD 400 mcg (turbo) od am	359	Randomized double blind multicenter	12 weeks parallel	**Lung function** FEV1 FVC **Symptoms** Change in symptoms % symptom free days use of rescue meds

Ukena *et al*	2003	CIC 400 mcg (mdi) od pm Vs. BUD 400 mcg (turbo) od pm	399	Randomized double blind multicenter	12 weeks parallel	**Lung function** FEV1 FVC, **Timing of onset of action** PEF **Symptoms** Change in symptoms % symptom free days use of rescue meds **HPA Suppression** Urine cortisol

Hansel *et al*	2003	CIC 100 mcg (mdi) od am Vs. CIC 400 mcg (mdi) od am Vs. BUD 200 mcg (turbo) bd	554	Randomized double blind multicenter	12 weeks parallel	**Lung function** FEV1 FVC PEF **Symptoms** Change in symptoms % symptom free days use of rescue meds **HPA Suppression** Urine cortisol

Derom *et al*	2001	CIC 400 mcg od Vs. CIC 800 mcg od Vs. CIC 800 mcg bd Vs. FP 500 mcg bd Vs. FP 1000mcg bd	25	Randomized double blind double dummy placebo controlled	7 days 6 period crossover with at least 3 weeks washout period	**Airway responsiveness to AMP** PC20 FEV1(doubling doses) **HPA Suppression** Plasma cortisol Urinary cortisol excretion

### Abstracts measuring lung function as end point

• Ukena *et al *reported significantly greater improvement in both FEV1 and FVC after CIC compared to BUD (P < 0.0001 and P = 0.0185 respectively).

• Hansel *et al *did not demonstrate any significant difference between CIC over BUD.

• Boulet *et al *reported superiority of CIC over FP for FVC (p < 0.01) but an insignificant difference for FEV1.

### Abstracts measuring patient symptoms as end point

• Ukena *et al *reported no significant difference in asthma symptom improvement between CIC and BUD, although CIC did demonstrate earlier onset of treatment effect (three days versus two weeks).

• Hansel *et al *did not demonstrate any significant difference between CIC over BUD

• Boulet *et al *reported no significant difference in asthma symptom changes between CIC and FP although the percentage of symptom free days was significantly higher in CIC vs. FP (43% vs. 43%, p = 0.0288)

### Abstracts measuring airway responsiveness to provoking agent as end point

• Derom *et al *reported no significant difference between CIC and FP for PC20 (AMP) FEV1

### Abstracts measuring HPA suppression as end point

• Ukena *et al *report no significant changes from baseline for urine cortisol levels for either CIC or BUD but do not compare the two treatments.

• Hansel *et al *report no significant changes from baseline for urine cortisol levels after CIC but a significant decrease after BUD. However they do not compare the two treatments.

• Derom *et al *report no significant changes from baseline for urine cortisol levels after CIC but a significant decrease after FP. However they do not compare the two treatments.

### Summary

None of the results reported in the abstracts challenge the conclusions of the review.

## Pre-publication history

The pre-publication history for this paper can be accessed here:


